# Impact of Conventional Cardiovascular Risk Factors on Left Internal Mammary Artery Graft Disease

**DOI:** 10.3389/fcvm.2021.705765

**Published:** 2022-01-14

**Authors:** Hui-Juan Zuo, Nan Nan, Hong-Xia Yang, Jin-Wen Wang, Xian-Tao Song

**Affiliations:** ^1^Department of Community Health Research, Beijing Anzhen Hospital, Beijing Institute of Heart Lung and Blood Vessel Diseases, Capital Medical University, Beijing, China; ^2^Department of Cardiology, Beijing Anzhen Hospital, Capital Medical University, Beijing, China

**Keywords:** coronary heart disease, coronary artery bypass grafting, left internal mammary artery, atherosclerosis, risk factor

## Abstract

**Background:**

The development of atherosclerosis was considered as the common cause of the stenosis of coronary artery grafts. Left internal mammary artery (LIMA) was the best artery graft for further effectiveness of coronary artery bypass grafting (CABG). We sought to assess the impact of known conventional cardiovascular risk factors (RFs) on LIMA graft stenosis.

**Methods:**

A retrospective study including 618 participants, who had recurrence of chest pain after CABG, aged ≥18 years, hospitalized for coronary angiography in Beijing Anzhen hospital between 2010 and 2017 was performed. All the participants were confirmed to have LIMA graft. Multivariate analysis was conducted to determine the relationship between conventional RFs and LIMA graft stenosis.

**Results:**

Of the study, 220 (35.6%) participants continued to smoke, 504 (81.6%) were overweight or obese, and 411 (66.5%) and 242 (39.2%) reported concomitant hypertension and diabetes, respectively. LIMA graft stenosis occurred in 161 participants (26.1%). Postoperative smoking, a CABG duration of ≥10 years and hyperglycemia without diabetes had an increased risk of LIMA graft stenosis, the odds ratio (OR) was 1.86 [95% confidence interval (CI): 1.26–2.78], 2.24 (95%CI:1.33–3.478), and 2.44(95% CI:1.39–4.32), respectively. Statin use (OR, 0.28; 95% CI: 0.25–0.5) and low-density lipoprotein cholesterol (LDL-C) < 1.8 mmol/L (OR, 0.27; 95% CI: 0.14–0.53) had a significantly decreased risk of LIMA graft stenosis. While, only 15.4% (95/618) achieved the target LDL-C level.

**Conclusions:**

Postoperative smoking and hyperglycemia without diabetes had an increased risk of LIMA graft stenosis. Statin use and LDL-C <1.8 mmol/L decreased the risk.

## Introduction

Coronary artery bypass graft (CABG) surgery is the most complete and durable treatment of left main coronary artery and three-vessel coronary severe stenosis or occlusion disease and has been an established therapy for nearly 50 years. Following CABG, effects should be done to reduce the recurrence of cardiovascular event. Studies have shown that the recurrence rate of angina is 17% in 1 year after CABG and 63% in 10 years after CABG (1), which was associated with native coronary artery disease progression and the development of graft atherosclerosis, and the stenosis of grafts was considered as the most common cause (2).

The grafts were saphenous vein (SV), left internal mammary artery (LIMA), right internal mammary artery (RIMA) and radial artery (RA). Using graft from LIMA to the left anterior descending artery (LAD) improves the effectiveness of CABG with high long-term patency rate (3). The great SV as a second conduit are commonly used for other target vessels of multi-vessel disease, however, they are associated high failure rate; the stenosis rate was more than 20% in 1 year, 40% in 2 years and 60% in 10 years after CABG (4). RA seems to be better graft than SV because prognosis improvement in increasing the long-term survival and decreasing the incidence of cardiovascular events were found in more and more studies (5–7). So, full arterial grafting for further effectiveness of CABG may be the direction (8, 9). Even though RA graft are strongly suggested in CABG surgery as the second artery graft (5–9), it appears to be underutilized in China with the percentage <1%. Hence, LIMA graft stenosis will be highlighted in this study.

The patency rate of grafts rapidly increases within the first year after CABG and then slowly,which was attributed to anastomotic site stenosis between 1 month and 1 year and atherosclerosis development of the bypass graft 1 year later (10). Dyslipidemia, hypertension, diabetes mellitus, obesity and smoking were considered as conventional risk factors (RFs) for atherosclerosis, which largely contribute to cardiovascular disease (CVD), approximately 70% of native coronary artery case and deaths were attributed to them (11). Are those conventional RFs associated with the development of atherosclerosis in artery grafts? Conflicting evidence was reported in previous studies (12–17). Therefore, we performed this study, enrolled patients who had recurrence of chest pain after performing CABG surgery at least 1 year, analyzed the impact of conventional cardiovascular RFs on LIMA graft stenosis.

## Methods

### Participants

This study was based on a retrospective, single-center analysis of patients who had recurrence of chest pain after CABG and had subsequent coronary angiographic findings in the Department of Cardiology of Beijing Anzhen Hospital from January 2010 to December 2017. All the participants were enrolled through the hospital's electronic record system. The inclusion criteria were an age of ≥18 years; recurrence of chest pain after CABG; completed electronic medical records and coronary angiographic findings; underwent LIMA-LAD graft. The exclusion criteria were CABG within 1 year before the start of the study; second CABG; renal insufficiency requiring dialysis treatment; and/or cancer.

### Data Collection

Data regarding the patients' demographics, conventional RFs for CVD, coronary angiographic findings and medication intake were collected from the hospital records by trained abstractors, using physician notes, laboratory reports, patient histories, and discharge summaries. A structured questionnaire was developed. Demographic information consisted of gender, age; conventional RFs included Overweight/obese, medical history of hypertension and diabetes, LDL-C level and smoking details. Basic medication therapy for secondary prevention to cardiovascular events included the usage of aspirin, β-blocker, angiotensin receptor blockers (ARB)/angiotensin-converting enzyme inhibitors (ACEI) and statins (dosage and type of those drugs were not obtained in the study). Height, weight, and blood pressure were measured in ward and recorded. The fasting plasma glucose (FPG) and LDL-C concentrations were examined while the patients were hospitalized. Coronary angiographic findings was reviewed, occlusion or stenosis of the native coronary arteries (including the left main, left anterior descending, circumflex, and right coronary arteries) and artery grafts were recorded. Number and stenosis of SV graft was not collected in this study.

### Measurements and Diagnostic Criteria

Chest pain was identified based on induction factor, position pain, feature of pain, pain duration, radiating pain and remission. We defined severe stenosis for native coronary artery as stenosis of ≥50% for left main, ≥70% for left anterior descending, circumflex, and right coronary arteries; defined artery graft disease as stenosis of ≥50%, and at least 1 stenosis was measure in LIMA based on angiography. Uncontrolled blood pressure was defined as SBP of ≥140 mmHg and / or DBP of ≥90 mmHg, and controlled blood pressure was defined as SBP of <140 mmHg and DBP of <90 mmHg. The body mass index (BMI) was calculated as body mass in kilograms divided by height in meters squared. Normal weight was defined as a BMI of <24.0 kg/m^2^, an overweight status was defined as a BMI of 24.0 to 27.9 kg/m^2^, and obese was defined as a BMI of ≥ 28.0 kg/m^2^. Ideal target of LDL-C was defined as an LDL-C concentration of <1.8 mmol/L (70 mg/dl). Ideal target of blood glucose was defined as an FPG of <7 mmol/L (126 mg/dl). The presence of hypertension or diabetes was defined by a previous diagnosis of hypertension or diabetes in the document of diagnosis on admission or summary of history. Nonsmokers were defined as patients who had never smoked, ever-smokers who stopped smoking at least 6 months, and current smokers were defined as those who were smoking every day or some days after CABG or stopped smoking <6 months at the time of interview.

### Statistical Analysis

The statistical analysis was performed with SPSS software for Windows, version 18.0 (SPSS, Inc., Chicago, IL, USA). Normally distributed continuous variables are presented as means ± standard deviations. A student's *t*-test was used to compare two independent samples. Categorical variables are expressed as proportions. The chi-square test was used to compare the differences in proportions between different groups. Multiple backward stepwise logistic regressions were performed to identify the association between risk factors and artery graft disease. All significant variables based on the chi-square test were entered into a multivariate model. All reported *P* values were two-tailed, and *P* < 0.05 were considered statistically significant.

## Results

### General Information

A total of 618 participants (male: *n* = 500, female: *n* = 118), 13 (2.1%) together with RIMA graft and 4 (0.65%) together with RA graft. The age ranged from 37 to 75 years (mean, 63.6 ± 8.8 years). Mean interval after CABG was 6.4 ± 3.5 years (range from 1 to 18 years), and 83.8% of them had undergone CABG <10 years prior to the study. The prevalence of conventional RFs was postoperative smoking 220 (35.6%), overweight or obese 504 (81.6%), hypertension 411 (66.5%), diabetes 242 (39.2%), and 15.4% (95 of 618) achieved LDL-C target level of <1.8 mmol/L. Recurrent angina was the most common presentation (93.4%, 577/618). According to the cut-off point of coronary artery stenosis, 298 (48.2%) patients had a left main disease, 473 (76.5%), 383 (62.0%), and 269 (43.5%) had a severe stenosis in LAD, circumflex coronary artery, and right coronary artery, respectively. LIMA graft stenosis occurred in 161 participants (26.1%). Main characteristics of the overall sample are presented in Table 1.

**Table 1:** General characteristics of the study population.

**General characteristics**	**Total subjects**
Sex, male	500 (80.9)
**Age (y)**	
Mean age	63.6 ± 8.8
<65	313 (50.6)
≥65	305 (49.4)
**Hospital in which CABG surgery underwent**	
Anzhen hospital	403 (65.2)
Other hospital	215 (34.8)
**Duration of CABG (y)**	
<5	236 (38.2)
5–9	266 (43.0)
≥10	116 (18.8)
**Conventional risk factors**	
Postoperative smoking	220 (35.6)
Overweight/obesity (BMI ≥ 24)	504 (81.5)
LDL-C ≥1.8 mmol/L	523 (84.6)
History of hypertension	411 (66.5)
History of diabetes	242 (39.2)
Prior MI	186 (30.1)
**Current presentation**	
Angina	577 (93.4)
Acute MI	41 (6.6)
**Native coronary artery severe stenosis**	
Left main stenosis	298 (48.2)
Left anterior descending stenosis	473 (76.5)
Circumflex coronary artery stenosis	383 (62.0)
Right coronary artery stenosis	269 (43.5)
LIMA graft stenosis	161 (26.1)

*Data are presented as mean ± standard deviation or n (%)*.

*CABG, coronary artery bypass grafting; BMI, body mass index; LDL-c, low-density lipoprotein cholesterol, MI, myocardial infarction; LIMA, internal mammary artery*.

### Mean Level and Optimal Control for Conventional RFs According to LIMA Graft Stenosis

Mean LDL-C level was 2.94 mmol/L among patients with LIMA graft stenosis, which was higher than that in those without LIMA graft stenosis (2.69 mmol/L, *P*=0.033). For mean level of SBP, DBP, FPG and BMI, no correlation with LIMA graft stenosis were observed (all p >0.05). Higher proportion of patients with LDL-C <1.8 mmol/L was observed in patients without LIMA graft stenosis than that in those with LIMA graft stenosis (18.2 vs. 7.5%, *P* = 0.001). There were significant differences in the proportion of FPG <7.0 mmol/L and smoking cessation across LIMA graft stenosis groups (*P* <0.05). There were no significant differences of in the proportion of BMI <24 kg/m^2^ and BP <140/90 mmHg between the two groups (Table 2).

**Table 2:** Mean level and the proportion of optimal control for conventional RFs according to LIMA graft stenosis.

**Conventional RFs**	**LIMA graft stenosis**		
	**No** **(***n*** = 457)**	**Yes** **(***n*** = 161)**	***P*** **value**
SBP (mmHg)	129 ± 18	129 ± 17	0.667
DBP (mmHg)	76 ± 11	76 ± 10	0.596
FPG (mmol/L)	5.87 ± 2.09	6.02 ± 2.41	0.453
LDL-C (mmol/L)	2.69 ± 0.91	2.94 ± 0.83	0.033
BMI (kg/m^2^)	27.1 ± 3.6	27.5 ± 4.3	0.250
BMI <24 (kg/m^2^)	86 (18.8)	28 (17.4)	0.688
LDL-C <1.8 (mmol/L)	83 (18.2)	12 (7.5)	0.001
BP <140/90 mmHg (in patients with hypertension)	172 (55.8)	55 (53.4)	0.666
FPG <7.0 mmol/L (in patients with diabetes)	93 (51.7)	23 (37.1)	0.048
Smoking cessation (in smokers at baseline)	88 (37.6)	12 (14.0)	<0.001

*Data are presented as mean ± standard deviation or n (%)*.

*RFs, risk factors; LIMA, left internal mammary artery; SBP, systolic blood pressure; DBP, diastolic blood pressure; LDL-C, low-density lipoprotein; BMI, body mass index; BP, blood pressure; FPG, fasting plasma glucose*.

### Correlation of Conventional RFs With LIMA Graft Stenosis

Of 618 patients, 161 (26.1%) developed LIMA graft stenosis, which did not vary by sex, age group, BP group, BMI group, and use of aspirin, ACEI/ARB and β-blocker (all *P* > 0.05). It was more frequent among patients without history of diabetes who had an FPG≥7.0 mmol/L, with postoperative smoking, or with a duration of CABG≥10 years (all P <0.05); lower prevalence was observed among patients with LDL-C <1.8 mmol/L and taking statin (*P* = 0.001). Multivariate logistic regression analysis was performed to identify the association between conventional RFs and LIMA graft disease. LIMA graft disease correlated with high blood glucose without diabetes (OR, 2.44; 95% CI: 1.39–4.32; *P* = 0.002), postoperative smoking (OR, 1.86; 95%CI: 1.26–2.78; *P* = 0.002) and duration of CABG≥10 years (OR, 2.24; 95%CI: 1.33–3.78; *P* = 0.003), LDL-C achieved target level (OR, 0.27; 95% CI: 0.14–0.53; *P* < 0.001) and statin use (OR, 0.38; 95% CI, 0.25–0.56; *P* < 0.001). Details are given in Table 3.

**Table 3:** Prevalence of LIMA graft stenosis and the relationship between LIMA graft stenosis and conventional RFs.

**Characteristics**		**Number**	**Prevalence of LIMA graft stenosis**		**Multivariate analysis**	
			***n*** **(%)**	***P*** **value**	**OR (95% CI)**	***P*** **value**
**Gender**						
Male		500	127 (25.4)	0.447		
Female		118	34 (28.8)			
**Age group (y)**						
<65		313	77 (24.6)	0.405		
≥65		305	84 (27.5)			
**Duration of CABG (y)**						
<5		236	55 (23.3)	0.021	1	
5–9		266	64 (24.1)		1.11 (0.72–1.72)	0.641
≥10		116	42 (36.2)		2.24 (1.33–3.78)	0.003
Blood pressure (mmHg)				0.196		
HP^−^ BP <140/90		185	48 (25.9)			
BP≥140/90		22	10 (45.5)			
HP^+^ BP <140/90		227	55 (24.2)			
BP≥140/90		136	48 (26.1)			
Blood glucose (mmol/L)				0.006		
DM^−^ FPG <7.0		303	70 (23.1)		1	
FPG≥7.0		73	29 (39.7)		2.44 (1.38–4.32)	0.002
DM^+^ FPG <7.0		116	23 (19.8)		0.84 (0.48–1.48)	0.539
FPG≥7.0		126	39 (31.0)		1.51 (0.92–2.47)	0.104
LDL-C (mmol/L)				0.001		
≥2.6		331	103 (31.1)		1	
1.8–2.59		192	46 (24.0)		0.63 (0.41–0.98)	0.041
<1.8		95	12 (12.6)		0.27 (0.14–0.53)	<0.001
BMI group				0.595		
<24		114	28 (24.6)			
24–27.9		199	48 (24.1)			
≥28		305	85 (27.9)			
**Postoperative smoking**						
No		398	87 (21.9)	0.001	1	
Yes		220	74 (33.6)		1.86 (1.26–2.77)	0.002
Aspirin use	No	117	36 (30.8)	0.197		
	Yes	501	125 (25.0)			
ACEI/ARB use	No	413	111 (26.9)	0.476		
	Yes	205	50 (24.4)			
Statin use	No	205	74 (36.1)	<0.001	1	
	Yes	413	87 (21.1)		0.28 (0.25–0.56)	<0.001
β-blocker use	No	185	41 (22.2)	0.150		
	Yes	433	120 (27.7)			

*OR, odds ratio; CI, confidence interval; CABG, coronary artery bypass grafting; HP^−^, patients without hypertension; HP^+^, patients with hypertension; BP, blood pressure; DM^−^, patients without diabetes; DM^+^, patients with diabetes; FPG, fasting plasma glucose; LDL-c, low-density lipoprotein cholesterol; BMI, body mass index; ACEI, angiotensin-converting enzyme inhibitor; ARB, angiotensin receptor blocker*.

### Statin Use and LDL-C Control

Of the study, 413 patients (66.8%) were taking statins, there was no significant difference in statin use among different duration of CABG, but it appeared to be lower in women, patients aged ≥65 years, and patients with LIMA graft stenosis (*p* < 0.05). In total, 95 patients (15.4%) achieved target LDL-C level, and the figure was 17.4% (72/413) in treated patients. Regardless of the treated patients or the overall sample, the frequency of achieving target LDL-C level of patients with LIMA graft stenosis was lower than that of patients without LIMA graft stenosis (Table 4).

**Table 4:** Statin use and LDL-C control of the study population.

**Characteristics**		**Statin use**	**LDL-C <1.8 mmol/L**	
			**Treated patients**	**All patients**
Sex^#^	Male	345 (69.0)	60 (17.4)	75 (15.0)
	Female	68 (57.6)	12 (17.6)	20 (16.9)
Age group (y)^#^	<65	227 (72.5)	42 (18.5)	46 (14.7)
	≥65	186 (61.0)	30 (16.1)	49 (16.1)
Duration of CABG (y)	<5	160 (67.8)	26 (16.3)	33 (14.0)
	5–9	173 (65.0)	34 (19.6)	46 (17.3)
	≥10	80 (69.0)	12 (15.0)	16 (13.8)
Artery graft disease^#^^§^	Yes	87 (54.0)	10 (11.5)	12 (7.5)
	No	326 (71.3)	62 (19.0)	83 (18.2)

# *Significant difference in statin use between the two groups (p < 0.05)*;

§ *Significant difference in LDL-C control between patients with or without artery graft disease*.

*LDL-C, low-density lipoprotein cholesterol; CABG, coronary artery bypass grafting*.

## Discussion

Our study indicated 26.1% of patients who had recurrence of chest pain after CABG developed stenosis in LIMA grafts. Postoperative smoking and hyperglycemia without diabetes had an increased occurrence of LIMA stenosis. Statin use and LDL-C <1.8 mmol/L were more common in patients without LIMA stenosis.

Strong evidence supports that conventional RFs including age, sex, hypertension, diabetes, total cholesterol, obesity, and smoking, which were widely used to estimate the risk of CVD based on the risk score system (18–20). Little was known whether all of them attributed to coronary artery grafts atherosclerosis development. In the present study, we identified long duration after CABG and postoperative smoking as a potential RF; we also demonstrated a novel association between hyperglycemia without diabetes and artery graft disease. Statin use and LDL-C <1.8 mmol/L were protective. Age, sex, hypertension, and obesity were not associated, which are quite difference from other studies. For example, Shah et al. found no patients variables (age, gender diabetes, hypertension) were significantly associated with graft patency in symptomatic patients after CABG (21). Conflicting evidence was found for each conventional RFs.

Hypertension is the largest risk factor with population attributable fraction (PAFs) of 22.3% for CVD (11). But it was not associated with artery graft stenosis in the present study, the prevalence of hypertension and mean level of SBP and DBP was similar between patients with or without artery graft stenosis, which was consistent with previous study (12, 13, 21). Individuals who had no history of hypertension with BP≥140/90 mmHg, it should be noted, had a high prevalence of artery graft stenosis, but the real associations might have been concealed because of the small sample size.

Diabetes is an important predictor of CVD (22), its influence on long-term patency of grafts is also important because nearly 30–40% of subjects undergoing CABG have diabetes (13–16). Conflicting evidence were reported in previous studies. Some identified diabetes as a potential predictor for graft failure (13), some showed negative association (21), and the rest showed that diabetes reduced the risk of LIMA graft stenosis (14–16). In the current study, four groups were divided according to the history of diabetes and the FPG level, hyperglycemia without diabetes had a higher prevalence of artery graft senosis than other groups, we than identified the association with OR = 2.44 (95%CI: 1.38–4.32). These individuals were suspected to be undiagnosed diabetes; they are more dangerous because most of them did not receive any hypoglycemic treatment and lifestyle intervention than previous diagnosis of diabetes. We now have a large population of undiagnosed diabetes with high percentage of 63% among patients with diabetes based on the latest national survey (23).

LDL-C lowering reduced the risk of recurrent ischemic cardiovascular events, guidelines on the management of blood cholesterol recommended a target LDL-C concentration of <1.8 mmol/L for patients with CVD to reduce recurrent risk (24) even <1.4 mmol/L for more benefit (25). Quin JA, et al. reported subjects who achieved a LDL-C target of <2.6 mmol/L at 1-year did not experience improved clinical outcomes or graft patency (26). A systematic review concluded that LDL-C lowering to 1.8 mmol/L may benefit post-CABG (27). The present study indicates that LDL-C <1.8 mmol/L had a low artery graft disease. However, the optimal target (LDL-C <1.8 mmol/L) is seldom achieved in patients with coronary heart disease (27). Strong evidence supports the benefit of statins use with respect to the risk of recurrent cardiovascular events and improve survival have largely been attributed to reduction of LDL-C (28). Statin therapy also reduces the inflammatory cascade and promotes stability of coronary lesions vulnerable to rupture. The present study indicates that statin use could reduce the longer-term risk of artery graft stenosis, even in subjects who didn't achieved the LDL-C target. Systematic review about statin therapy before and after CABG concluded, postoperative statins reduce the recurrence of cardiovascular events (27).

Although some cross-sectional studies did not show the association between postoperative smoking and coronary artery graft lesions (13), follow-up studies showed smoking cessation turned out to have a great effect on reducing angina pectoris or long-term mortality, the effect even greater than that of any other intervention or treatment (16, 17, 29, 30). Positive association was also found in this current study. Therefore, patients with CHD, especially those undergoing CABG should be encouraged to stop smoking. Previous studies showed 43–68% of patients undergoing CABG stopped smoking (29, 30). Continuous smoking cessation intervention is needed for patients care after CABG.

The major limitation of this study is a single center and retrospective analysis, all the information was absorbed from the hospital electronic medical records, detail information about diet, physical inactivity and dosage of medication was not collected, therefore it is likely that there is a selection and recall bias related to the inquiry of medical history and the structured questionnaire. The sample size of the present study was also limited. In addition, we aimed to evaluate the effect of traditional RFs for CVD on artery graft stenosis, other RFs (13) that might be associated with graft stenosis were not collected and unadjusted, such as plasma fibrinogen, creatinine, and lifestyles. Glycosylated hemoglobin level was not collected because of high percentage missing value in the electronic medical record, or undiagnosed diabetes could be identified.

## Conclusions

This study provides more evidence for the RFs of coronary artery grafts artery, not all conventional risk factors were positively associated with artery graft stenosis. Long duration after CABG and postoperative smoking were identified as a potential risk factor, hyperglycemia without diabetes, it should be noted, had a high prevalence of artery graft stenosis. Statin use and LDL-C <1.8 mmol/L might be protective. The results will help clinicians to take both lifestyle and pharmacological interventions to reduce artery graft lesions risk.

## Data Availability Statement

The original contributions presented in the study are included in the article/supplementary material, further inquiries can be directed to the corresponding authors.

## Ethics Statement

The studies involving human participants were reviewed and approved by the Institutional Review Boards of Beijing Anzhen Hospital, Capital Medical University, Beijing, China. Written informed consent for participation was not required for this study in accordance with the national legislation and the institutional requirements.

## Author Contributions

H-JZ and X-TS conceived and designed the study. H-JZ and H-XY prepared the database, performed the analysis, interpreted results, and drafted the manuscript. NN and J-WW prepared the database. X-TS interpreted results and performed critical revision of the manuscript. All authors read and approved the final version of the manuscript.

## Funding

This research received a research grant from Beijing Municipal Science and Technology (No. Z16110000016139).

## Conflict of Interest

The authors declare that the research was conducted in the absence of any commercial or financial relationships that could be construed as a potential conflict of interest.

## Publisher's Note

All claims expressed in this article are solely those of the authors and do not necessarily represent those of their affiliated organizations, or those of the publisher, the editors and the reviewers. Any product that may be evaluated in this article, or claim that may be made by its manufacturer, is not guaranteed or endorsed by the publisher.

## Abbreviations

RFs: risk factors
LIMA: left internal mammary artery
CVD: cardiovascular disease
CABG: coronary artery bypass grafting
SV: The grafts were saphenous vein
RIMA: right internal mammary artery
RA: radial artery
LAD: left anterior descending artery
OR: odds ratio
CI: confidence interval
LDL-C: low-density lipoprotein cholesterol
ARB: angiotensin receptor blockers
ACEI: angiotensin-converting enzyme inhibitors
BMI: body mass index.
